# Understanding the complexity of surgical decision-making for individuals with symptomatic lumbar spinal stenosis: A qualitative study

**DOI:** 10.1186/s12998-025-00622-y

**Published:** 2025-11-26

**Authors:** Nora Bakaa, James Gillet, Raja Rampersaud, Brian Drew, Aleksa Cenic, Lisa Carlesso, Joy MacDermid, Douglas P. Gross, Joanne Thorne, Luciana G Macedo

**Affiliations:** 1 Department of Rehabilitation Sciences, McMaster University, 1400 Main St. W, Hamilton, Ontario Canada; 2 Faculty of Social Sciences, McMaster University, Hamilton, Ontario Canada; 3 University of Toronto, Toronto Western Hospital, 399 Bathurst St, Toronto, Ontario Canada; 4 Department of Surgery, McMaster University, Hamilton, Ontario Canada; 5 School of Physical Therapy, University of Western Ontario, London, Ontario Canada; 6 Department of Physical Therapy, University of Alberta , Edmonton, Alberta Canada; 7 Patient Partner, Hamilton, Ontario Canada

**Keywords:** Lumbar spinal stenosis, Qualitative, Decision-making, Phenomenology

## Abstract

**Background:**

Understanding the factors influencing surgical decisions specific to symptomatic lumbar spinal stenosis (SLSS) can help healthcare providers support patients, set expectations and improve health literacy. Therefore, the purpose of this study is to explore the experiences and perspectives of Canadian patients who choose to undergo surgery for SLSS.

**Methods:**

We used qualitative interpretive phenomenology to understand the surgical decision-making process from individuals with lived experience of SLSS. We conducted semi-structured qualitative interviews that lasted between 30 and 90 min. Inclusion criteria were individuals 55 or older, diagnosed with SLSS, scheduled for or have undergone lumbar spine surgery, and able to speak English. Participants were recruited between October 2019 and September 2021.

**Results:**

A total of 32 participants (Men: *n* = 18; Women: *n* = 14) were included in this study. Among those participants, 15 were interviewed preoperatively and 17 postoperatively, and all were over 55 years. We identified 5 themes that were woven through the decision-making of respondents, beginning with the experience with healthcare systems and building outwards to the broader social context: (1) Previous experience with non-surgical management, (2) Worrisome symptoms impacting functionality, (3) Perception of surgery as a final course of action, (4) Post-surgical hopes/expectations (i.e., hope that they will be pain-free after surgery), and (5) Having a social support network (i.e., advice and support from family/friends).

**Conclusion:**

Several experiences may influence an individual’s decision to undergo spine surgery, highlighting the importance of integrating a biopsychosocial model in managing SLSS. For chiropractors and manual therapists, these indicators are particularly important, as they often represent a first point of contact for patients with SLSS. Clinicians must maintainperson-centric communication to help patients understand their condition and the clinical treatment pathway for SLSS and develop post-surgical expectations.

**Supplementary Information:**

The online version contains supplementary material available at 10.1186/s12998-025-00622-y.

## Key points

1) Surgical decision-making is a complex experience that requires both a biomedical and psychosocial approach.

2) Improved shared decision-making among patients and healthcare providers may improve patient expectations.

3) There is a need to develop resources and standardize pathways related to managing symptomatic lumbar spinal stenosis.

**Supplementary Information:**

The online version contains supplementary material available at 10.1186/s12998-025-00622-y.

## Introduction

Symptomatic lumbar spinal stenosis (SLSS) is a prevalent degenerative spinal condition that poses a significant burden on older adults and healthcare systems worldwide [1]. Neurogenic claudication is the most common symptom of SLSS, often described as pain, cramping, tingling, numbness, and/or weakness in the gluteal region or leg/s, that is exacerbated by walking or standing and relieved by lumbar flexion [2–4]. Standardized treatment pathways for SLSS have been developed to improve the care of patients with SLSS [5]. Non-surgical management, including exercise, manual therapy, and injections, is considered the first line of care, with surgical interventions recommended with persistent severe symptoms or when non-surgical management has failed [1].

In Canada, approximately 30,000 Canadians undergo spine surgery annually, [6] which is expected to increase [7]. For those who do not recover spontaneously or benefit from non-surgical management, surgery is recommended. However, little is known about how patients proceed with SLSS surgery. To our knowledge, only two qualitative studies have explored the surgical decision-making process in this population [8, 9]. One study in Hong Kong found that decisions were influenced by surgeons’ recommendations, patient-surgeon relationships, symptoms, treatment history, social influences, and expected outcomes [8]. Another study on patients with sciatica showed a disconnection between what surgeons articulated and what patients understood [10]. Understanding the factors influencing surgical decisions specific to SLSS can help healthcare providers support patients, set expectations and improve health literacy. Therefore, the purpose of this study is to explore the experiences and perspectives of Canadian patients who choose to undergo surgery for SLSS.

## Methods

*Research paradigm and design*: The study was grounded in a constructivist paradigm and guided by interpretive phenomenology, [11] which seeks to understand how individuals make sense of their lived experiences through the interpretation and co-construction of meaning between researcher and participant. This approach was well-suited to our aim of exploring how individuals with SLSS interpret and give meaning to their experiences, expectations, and emotions surrounding surgical decision-making and perioperative care, recognizing that understanding is shaped by both personal and contextual factors. The Standards for Reporting Qualitative Research (SRQR) guidelines were followed [12]. 

*Setting/Participants* : Participants were recruited from the surgical practices of participating orthopaedic surgeons at the University Health Network in Toronto and Hamilton General Hospital in Hamilton, Ontario. To ensure a diverse range of participants, particularly in age and sex, purposive sampling was employed to recruit eligible patients. Participants were eligible for inclusion if they met the following criteria: (1) 55 years of age or older, (2) diagnosed with SLSS who were scheduled for surgery or previously had surgery at least 6 months before the study, and (3) ability to speak English. Out of the 55 participants eligible for inclusion in the study, 40 agreed to be contacted for an interview. Subsequently, all 40 participants were contacted by phone, and 35 consented and completed the interview; of those, 32 (15 preoperatively and 17 postoperatively) were included in this analysis (3 had technical issues with the transcript recordings and were excluded from this study).

*Recruitment*: The research team contacted individuals who met the eligibility criteria via a letter sent through the mail. The letter included a written explanation of the study’s objectives and the role of interested participants. JG contacted interested participants to schedule the interview and obtained written consent. Participants were recruited between October 2019 and September 2021.

*Researcher characteristics*: Interviews were conducted by JG, an associate professor in the faculty of social sciences with significant experience in qualitative research. Data were analyzed by NB, a chiropractor with clinical experience in treating SLSS, who holds a PhD in rehabilitation sciences with experience in qualitative research design. Both researchers had no prior relationships with any of the participants and acknowledged the importance of engaging in reflexivity throughout the study. While experience informed the interpretation of the study results, JG and NB remained committed to minimizing bias through thoughtful discussions.

*Data collection*: The interviewer (JG) conducted in-depth semi-structured phone interviews after obtaining the participants’ informed consent. A semi-structured interview guide (Appendix A), developed by JG, LM, and LC, was utilized to direct the conversations [13]. The interview guide was designed to understand participants’ reflections on how they came to the decision to undergo surgery, specifically, the personal and social factors shaping their choices, and the meanings they attached to those decisions. Questions were open-ended and exploratory (e.g., “Tell us about your decision to use surgery as a means of treating LSS”), encouraging participants to express lived experiences in their own terms [14]. This aligns with interpretive phenomenology’s emphasis on understanding experience as situated and relational. The flexibility of the guide allowed for a conversational, co-constructed process that allowed participants’ own narratives to guide the depth of inquiry. Before the end of the 45- to 90-minute interview, participants were asked to clarify any points or add anything that had not been previously addressed. All interviews were digitally audio-recorded, and identifying information was removed from the transcripts to ensure confidentiality. All interviews were transcribed verbatim, with participants’ names replaced by unique numbers. Interviews were conducted until sufficient information and interpretive depth were achieved, as determined by the research team (JG, LM, LC).

### Data analysis

Using interpretive phenomenology, our analysis aimed to understand the perceptions and experiences of the surgical decision-making process among individuals with SLSS, acknowledging that meaning is co-constructive and iterative [15]. Braun and Clarke’s [16–18] method for reflexive thematic analysis was performed to develop themes associated with the surgical decision-making process for older adults with SLSS. Reflexive thematic analysis aligns with interpretive phenomenology by emphasizing the subjective role of the researcher in constructing themes that represent shared and divergent meaning from participants. The qualitative software *Dedoose* [19] was used to manage, store, and code the data from each transcript. An inductive approach to thematic analysis was used to facilitate the coding of the data by the responses rather than preconceived theoretical or analytical knowledge.

The six-phase method included familiarization with the data, the initial generation of codes, searching the codes for potential themes, reviewing the themes, defining and naming the themes, and finalizing the results. Coding and theme development were flexible and interpretive; researchers engaged in iterative reading, coding and discussion to deepen understanding of the data. Initially, NB and JG completed a line-by-line reading of each transcript. NB constructed initial code names and definitions based on data from the transcripts, which were discussed with JG, LM, RR, and LC. Relevant codes that demonstrated a consistent pattern and a clear distinction from other themes were combined to form a theme by two research members (NB and LM). Each theme was iteratively analyzed and refined to construct a clear definition and name. The entire research team reviewed and refined themes and associated definitions. Direct quotes from each transcript were extracted to illustrate the key features of each theme.

*Methodological rigour*: To ensure methodological rigour and trustworthiness of the data, several well-established techniques were used [20]. Participant feedback (member reflection) was sought to deepen interpretive understanding of the constructed themes. Only one participant (JT) responded to feedback requests and subsequently served as a patient partner for this work. Researchers (NB, JG) maintained reflexive journals to critically consider how their perspectives and experiences may have shaped interpretation. Themes were discussed collaboratively to enhance reflexive interpretation and conceptual clarity. And lastly, an audit trail of decisions made throughout the study was maintained.

## Results

**Fig. 1:**
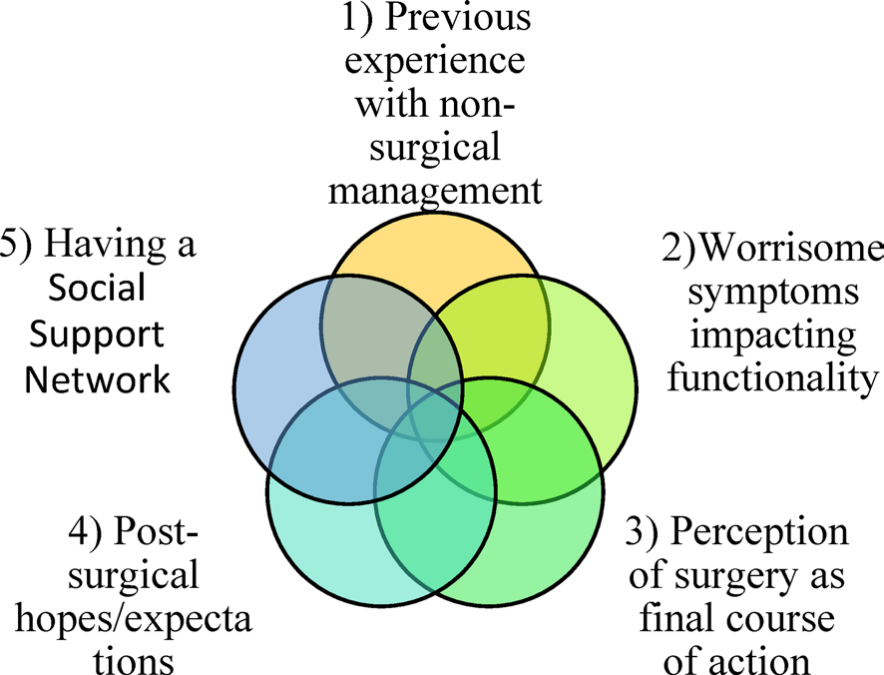
Overlapping themes associated with the surgical decision-making process for individuals with SLSS who have chosen to proceed with surgery

Of the 32 participants (Men: *n* = 18; Women: *n* = 14), all participants were over the age of 55 and were scheduled for SLSS surgery (*n* = 15) or had undergone SLSS surgery (*n* = 17) (Table 1: Participant Characteristics). We identified five overlapping themes, beginning with experiences with health care systems and building outwards to social experiences: (1) Previous experience with non-surgical management, (2) Worrisome symptoms impacting functionality, (3) Perception of surgery as the final course of action, (4) Post-surgical hopes and expectations, and (5) Having a social support network (Fig. 1). A comprehensive table of quotes fitting under each theme can be found in Table 2.

**Table 1:** Participant characteristics

Participant ID	Operation time	Sex
4	Postoperative	Male
5	Postoperative	Female
10	Postoperative	Female
14	Postoperative	Male
16	Postoperative	Male
17	Postoperative	Male
18	Postoperative	Female
20	Postoperative	Male
21	Postoperative	Male
22	Postoperative	Male
23	Postoperative	Male
24	Postoperative	Female
25	Postoperative	Male
26	Postoperative	Male
27	Postoperative	Male
29	Postoperative	Male
30	Postoperative	Male
2	Preoperative	Male
3	Preoperative	Female
7	Preoperative	Female
9	Preoperative	Female
11	Preoperative	Female
12	Preoperative	Female
13	Preoperative	Male
15	Preoperative	Female
31	Preoperative	Female
33	Preoperative	Female
34	Preoperative	Male
35	Preoperative	Female
36	Preoperative	Male
37	Preoperative	Male
40	Preoperative	Female

**Table 2:** Sample quotes for each theme

Themes	Quotes
Previous experience with non-surgical management	• P031: Physio didn’t help me. Chiropractor didn’t help me. Acupuncture, massage, nothing helped me. And then I finally got into the clinic, and they did an MRI. And of course, whatever’s in between your spine there is degenerative. And I have spinal stenosis too. • P031: And went through physio and chiropractor. And eventually after a couple of months, it went away, and I was okay. So, I’ve had a few since then. I’ve had a few issues that if I overdid it, that I would get the sciatica. But it would last a month or so, and then it would go away. But this time it didn’t. So this time was different. And this has been going on for almost two years now. • P010: I perceived back surgery to be a big stinking deal, I went into it with an idea that I wanted to know as much as I possibly could, and I wanted to know how to avoid surgery if at all possible. What are my other options, et cetera, et cetera. I did not find that there was a lot of support for that approach. • P007: So, I done number of the exercise which will help you to reduce your pain. And actually, it benefited me initially. And then I get through this exercise, I do morning and evening, and I think it takes half an hour at the time. So, morning half an hour and evening half an hour. So, one hour I do this bootcamp exercise. • P002: We’re just doing this [weight loss program] on our own. My wife and I are doing it. [My surgeon] basically said before the weight loss won’t make a lot of difference, but I’m doing it just to be in better shape to go into this.”
Worrisome symptoms impacting functionality	• P016: And I was at a fair amount of pain myself. Like, in the mornings I couldn’t stand up straight. I mean, I was just hobbled over like a real old man. And you couldn’t stand up straight, but after maybe by 11 o’clock and a bit of movement, and walker out, I can eventually straighten up. But I was in pain. So, I mean, look. While there might have been the odd little concern. I mean, the overriding view was I mean the balance was very much tipped in favor of “Let’s do it. Let’s get it done.” • P017: I couldn’t stand up straight. The pain was going down my buttocks and down into my leg. Once that happens, from what I’ve learned through this process is, that means the nerve is being damaged to a point where it may not come back from it. And he mentioned that to me, pre-surgery, he mentioned that he was going to try to straighten it out, and try it and see what it looked like when it was in there, because it was impinged, right? • P015: Because of the numbness in my leg and the fact that was where it seemed to me that the pain was radiating from the muscle in my leg. And also, when I tried to stand up it seemed to me the buttock area wasn’t kind of holding me up.
Perception of Surgery as Final Course of Action	• P002: The first place is that the pain isn’t there all the time, but when you stand for a very short time, or walk a very short distance, you get the pain that radiates down your leg, and it has like a numbness. So, it’s not getting any better. And according to [my surgeon], there’s not a lot you can do to help that along on your own. • P015: But just looking myself at what the MRI was showing and what was said in terms of the stenosis and the whatnot. It seems to me as I’m getting older that the situation will be getting worse. • P040: And that was one of the reasons, now that I’m looking at that, one of the reasons why I decided to go with a surgery and not kind of wait because he said it would be sooner rather than later. And so, I did say to him, when I was contemplating the fact that he had said that he would do it quickly. […] And to me that spoke that it was serious enough that it needed to be done sooner. • P016: My own tendency has always been “Stay away from doctors, hospitals. You can do this yourself. You can get better yourself. The body tends to heal itself.” But in this particular case, I guess, in my case anyway, I was getting to the point where it was no fun.
Post-surgical hopes/ expectations	• P003: But the reason that I didn’t even hesitate to support in all of this for sure. And supportive of this decision, you know? • P009: So, my best friend’s wife is a nurse practitioner, so she has a lot of knowledge and a lot of feedback for me. She also had a lot of consider it is the fact that when I first saw him, he said to me, “Can you live with the pain for the rest of your life?” And I said, “No, I can’t.” • P009: Well, what I’m hoping for is that I don’t have the pain any longer, but that may be… I’m also prepared for it not to be that good, but certainly better and more manageable than it is at its worst point. If that makes any sense. • P012: Just to not be in the pain that I’ve been going through, and not having to take a medication every day.
Having a Social Support Network	• P040: And so, my friend and I went for a coffee and talked about it, and she said to me, I don’t think you really have much of a choice. Like she said, my pain isn’t going to get any better. It might stay the same or like get worse. • P016: My wife is very supportive on all stuff. And she was supportive on this as well. And I’ve two sons and they are also in the same boat. Although, they don’t live nearby, but we communicate a fair bit. So, nobody said, “Oh, don’t do this” or anything like that. They were all well aware of what was going on and supportive. I mean, my two sons, they are fully involved with their own lives and their own kids and so on and so forth. But my wife was certainly a real contacts who she could sit down and give them all the details of what I was going through and get options and feedback. I was using that against what my doctor was telling me. For the most part, they were bang on. So, I felt good about that. It was kind of like a second opinion, I guess. I was looking at it that way anyway.

### Experience with non-surgical management

It was clear that participants considered non-surgical management the first-line approach to managing their condition. Their lack of success with non-surgical management was often a primary consideration for their decision to have surgery. Many participants described trying several treatment options, including chiropractic care, physiotherapy, acupuncture, and massage, among others, experiencing very little relief from their symptoms.

*P003: “I have tried all those things when I first had the pain that would not go away. I tried all those things. I went to a physio for so long, I did exercises, I did everything. And so, it came right down to the fact that this pain was not going away.”*

Some participants explained that non-surgical interventions seemed to aggravate their symptoms, while others described an initial good response followed by less improvement over time:

*P031: “[I] went through physio and chiropractor. And eventually after a couple of months*,* it went away*,* and I was okay. So*,* I’ve had a few since then. I’ve had a few issues that if I overdid it*,* that I would get the sciatica. But it would last a month or so*,* and then it would go away. But this time it didn’t. So*,* this time was different. And this has been going on for almost two years now.”*

There was a general sense of frustration around non-surgical interventions that did not seem to offer any relief of their symptoms, ultimately leading to their decision to have surgery:

*P012: “I understand everything to be a waste of time. I’ve done everything from acupuncture*,* to cupping*,* to massage to this*,* done and nothing seemed to work. So*,* I didn’t bother taking their suggestion going through physiotherapy [pre-operatively] because I knew that my surgery was coming up anyway and I’d probably be going through a physio after that.”*

It was important to note that there needed to be more consistency in the management of SLSS, as some participants described little to no experiences with non-surgical management. One participant describes being in a state of shock at being referred to surgery, given they had no previous recommendations for non-surgical options:

*P040: “I thought [the surgeon] was going to suggest I could do some alternative things, acupuncture, stuff like that, physiotherapy, or maybe there was a different kind of medication I could take or something like that. I had no idea that I was going to see him for surgery. […] He took me off guard when he started talking that I would be having surgery. When I was hesitant, he said, yes, I could go and think about it.”*

Some participants also described being told by their healthcare providers that interventions such as weight loss programs or exercise would be insufficient for managing their condition:

*P009: “But in terms of doing core exercise*,* what I was told was don’t do exercise or anything that hurts. So*,* the only exercises I would do would be mostly stretching*,* either hanging from a bar or stretching belly down*,* to touch your head that type of deal*,* and only to a certain point*,* depending on how much pain was involved."'*

Participants described a lack of consistency surrounding the non-surgical management of SLSS. To ensure patients receive appropriate care at the appropriate time, healthcare pathways for the management of SLSS need to be improved.

### Worrisome symptoms impacting functionality

Participants painted a vivid image when describing the impact of their condition on their physical capability and overall quality of life. One of the main contributing factors to their decision to have surgery was the leg-dominant symptoms that limited their ability to walk:

*P002: "But as far as the back pain goes*,* I would say that the pain isn’t as bad as the results of the pain*,* like the discomfort in the leg. Basically*,* a numbness that goes right into your groin and you can’t walk. It doesn’t hurt. It doesn’t feel like you’re going to fall*,* but you just don’t have any feeling in that leg*.”

The same participant states that the ideal post-operative outcome would be resolving the leg-dominant symptoms. Moreover, the terms (e.g., pain/numbness) used to describe their symptoms were often used interchangeably:

*P002: *“*The first place is that the pain isn’t there all the time*,* but when you stand for a very short time*,* or walk a very short distance*,* you get the pain that radiates down your leg*,* and it has like a numbness.”*

Participants often portrayed a significant amount of fear and anxiety associated with their symptoms. These feelings were emphasized when participants described the effects of their symptoms on their ability to walk, compounded with the fear that they may never be able to walk again:

*P012*: “*It just weighs on my mind*,* just sometimes I feel as if my legs aren’t working*,* and sometimes my legs go numb as I’m out for a walk. I feel like I’m walking*,* like I’m drunk; I probably am. Sometimes*,* I have to lean on something until I can position myself to get that nerve off of whatever it’s hitting; that’s making it do that.”*

Participants characterized pain as more than just physiological changes but rather the psychosocial aspects that altered their pain perception. For instance, one participant (*P040*) described feeling like their body was giving up on them:

*“It started, really, I don’t have pain where he says he’s going to operate. Three, four, maybe five, according to the MRI report. What I was feeling was like my leg muscles, my thigh muscles and the back of my legs were shrinking on me.”*

Participants emphasized that the back pain associated with SLSS was manageable, but the resulting leg-dominant symptoms led to significant fear and anxiety. Participants’ decision to have surgery was often driven by the hope of restoring the normal functioning of the leg/s—which is a critical clinical indicator for surgeons when considering eligibility for surgery.

### Perception of surgery as the final course of action

Surgery was considered the participants’ final course of action for managing their condition, given failed non-surgical management and the debilitating leg-dominant symptoms. Pain was often attributed to the specific pathoanatomical changes observed in their spine (e.g., degeneration). One participant (*P009*) recalled being given several treatment options to manage their condition but perceived that surgery was necessary due to the underlying pathoanatomical changes:

*“Well, after the MRI, probably six or seven weeks after I actually did it, I was called in to have a conversation with the surgeon. He started to give me my options. Some of which was, again, different medications and whatever, but ultimately, what he told me was this probably is not going to heal itself or would be recurring no matter what I do just based on what the actual issue was.”*

Similarly, another participant *(P002)* perceived surgery as the only choice as it was the only way to prevent their condition from worsening:

*“[The surgeon] showed me the results on the computer in his office before we had the set up for the operation and showed me how bad it was and explained thoroughly what he was going to do. And I thought, well, if I don’t then, my spine was coming in on my spinal cord. And if it kept up at the rate it was going, I would be in a wheelchair, I’d be crippled. Because it would’ve cut my spinal cord. So, I mean, that was a no brainer. I may be old, but I’m not stupid.”*

### Post-surgical hopes/expectations

Participants expressed different hopes and expectations regarding what life would be like after surgery. Most participants described significant limitations, both physically and socially, due to SLSS. There was a general hope among participants for a reduction in pain and disability to improve their quality of life after surgery. Some participants understood that the numbness/tingling they experienced in their leg would subside immediately after surgery, while others recognized that these symptoms would take time to heal: *P007*: “*I was told that once the surgery is done*,* the pain will go away immediately. But I know that tingling and numbness*,* it will take time and it may not go. So*,* it is okay for me. As long as pain is not there*,* I’m good*.”

Most participants described that the ideal outcome after surgery would be to regain their ability to walk pain-free:

*P011: *“*I would want to be able to resume walking distances without pain. I’d like to be able to turn in certain ways without getting a jab in my leg. And that would mean being able to play golf at a reasonably high level again. I’d like to be able to do odd jobs around the house and fix things without having to sort of crawl around.”*

One of the main functional expectations after surgery was the ability to walk again. Participants highlighted the desire to return to activities of daily living (e.g., playing golf and completing household tasks). Most participants viewed surgery as the final hope of returning to a life without pain or limitations. However, others expressed more subdued and realistic expectations:

*P009: “Well,what I’m hoping for is that I don’t have the pain any longer, but that may be… I’m also prepared for it not to be that good,but certainly better and more manageable than it is at its worst point. If that makes any sense.”009:*

Ultimately, participants hoped that surgery would allow them to regain their quality of life by reducing their pain and disability.

### Having a social support network

Social support was described as an essential component of the surgical decision-making process. Considering the advice of friends and family throughout their surgical journey helped to inform their decision, even if they did not take their advice. One participant explained how advice from a friend who had similar symptoms and had undergone surgery helped them make their decision:

*P003*: “*Well*,* because [a friend] had the very same symptoms and problems that I did. […] And he said*,* “No*,* it was the only option because there was no other … otherwise you have to live on pain pills*,* and you’re limited to what you can do.” So*,* there’s no two questions about it*.”

In another lens, one participant recalls getting advice from friends that they should not undergo surgery but decided to continue despite their advice:

*P015*: “*Well*,* different people have said different things to me*,* “don’t let them touch your back*,* don’t let them go in there. The chances of whatever if they dive into that*,* etc. Well*,* for me*,* life is risky. I’m hoping for the best. I’m hoping that they’ll go in*,* and they’ll scrape what needs to be scraped or release what needs to be released. And I will do whatever it is to follow up that’s necessary and my part to follow up. You can’t live thinking the worst and whatever.”*

Participants weighed various opinions before deciding to have surgery, and having a support network helped not only with their decision to have surgery but also their physical limitations.

## Discussion

Surgical decision-making for SLSS is a complex journey, with participants describing several experiences. These experiences began with the healthcare system and extended to include psychosocial influences. The identified themes included prior experience with non-surgical management, the worrisome symptoms of their condition that impact their function, their perception of surgery as a final course of action, their post-surgical hopes and expectations, and the importance of social support. Surgical decision-making requires a collaborative approach from healthcare providers, family, friends or caregivers, and patients [21]. To make informed decisions about surgery, participants use their prior experiences managing and coping with their condition and social support.

Most participants described ineffective non-surgical management as a driver for surgery, while others had no prior alternative management. These findings align with the literature, highlighting the need for standardization of non-surgical management for SLSS and the lack of uptake of developed management pathways [5]. Efforts include a recent Delphi study outlining a coordinated pathway to care [5] and Canada’s Rapid Access Clinics, which aim to reduce unnecessary imaging and surgical referrals [22]. Despite evidence-based guidelines for spinal pain, [23] SLSS management remains challenging due to inefficiencies in the navigation of the siloed Ontario healthcare system and utilization of treatment options with a lack of scientific evidence [24]. Implementing integrated care pathways may improve the management of SLSS but requires careful consideration and healthcare resources.

Participants in our study used various terms to describe their symptoms. Previous research showed that patients with SLSS commonly used pain and discomfort to explain their condition and less often mentioned tingling, weakness, burning paresthesia, or heaviness in the legs [25]. In this study, participants used several descriptive terms (e.g., pain, discomfort, weakness, numbness), sometimes interchangeably. The decision to undergo surgery was driven by fear and anxiety about leg-dominant symptoms and perceived worsening. These findings align with pain perception theories that suggest pain is influenced by cognitive and emotional inputs [26]. The implementation of a biopsychosocial model for the management of SLSS may help patients better understand their pain.

Participants had conflicting expectations of surgery, with some expecting a complete reduction in pain and others seeking a reduction in leg numbness and tingling. Understanding SLSS symptoms may help patients set realistic expectations (e.g., resolution of neurogenic claudication symptoms post-surgically, but not necessarily full symptom resolution, such as constant back pain or neuropathic symptoms), which may help patients recover [27–29]. SLSS often includes both radicular and neurogenic pain, [30] highlighting the need for resources that explain condition/symptom-specific symptoms to support patient understanding and recovery.

Participants described their condition through a biomedical lens, attributing pain to the pathoanatomical changes in the spine (e.g. disc degeneration) and seeing surgery as the only viable option for management. Participants inaccurately described the pathoanatomical changes in their spine, indicating a lack of understanding of surgeons’ explanations, consistent with previous literature [8–10]. The attribution of SLSS to predominantly pathoanatomical changes leads to a narrow understanding of pain perception, influencing post-operative recovery. Studies show that a biopsychosocial approach, compared to a biomedical lens, improves pain catastrophizing and the perception of their condition [31]. Given that pain catastrophizing and fear avoidance predict poor outcomes, [32] providing more education may help improve outcomes.

A social support network was an important influence in participants’ decision to undergo surgery. Consistent with previous research, [8, 33] our study highlights the psychological benefits of involving family and friends in forming opinions on undergoing surgery. The emotional support systems and shared decision-making highlight the psychosocial drivers in the provision of comprehensive management of SLSS [34–36]. Embracing shared decision-making improves collaboration between patients, social support networks, and healthcare providers and acknowledges the biopsychosocial model in addressing pain. This is particularly important for rehabilitation providers (e.g., chiropractors, physiotherapists, and other manual therapists) as they are often the first line of treatment for individuals with SLSS.

## Strengths/limitations

This study has several limitations. Participants who consented may have different decision-making perspectives than those who declined. Interviews were conducted pre- and post-surgery, but experiences may differ, with pre-surgical participants having higher pain levels and post-surgical participants facing recall bias. We did not interview surgical candidates who opted out, limiting insights to non-surgical coping strategies. Although limited to surgical patients, their descriptions provided broader insights into decision-making. Future studies should aim to interview patients before and after their consultation with the surgeon to better understand why some patients choose surgery and others do not.

## Conclusion

In conclusion, this study found that several experiences may influence an individual’s decision to undergo spine surgery, highlighting the importance of integrating a biopsychosocial model in the management of SLSS. For chiropractors and manual therapists, these indicators are particularly important, as they often represent a first point of contact for patients with SLSS. The complexity surrounding surgical decision-making suggests the need to develop comprehensive approaches for the management of SLSS.

## Supplementary Information

Below is the link to the electronic supplementary material.

Supplementary Material 1

## Data Availability

The data supporting this study’s findings consist of qualitative interviews and transcripts, which are not publicly available due to privacy and confidentiality concerns. Participants consented to use their data for research purposes under the assurance that their identities and personal information would remain protected. As such, sharing raw data publicly is not feasible.
