# Supplementary material for: Understanding the complexity of surgical decision-making for individuals with symptomatic lumbar spinal stenosis: A qualitative study
Source: Chiropr Man Therap. 2025 Nov 26;33:58. doi: 10.1186/s12998-025-00622-y (PMC12751202; doi:10.1186/s12998-025-00622-y)
Supplement: Supplementary file 1 — Supplementary Material 1 [file 12998_2025_622_MOESM1_ESM.docx]

## Appendix A: Semi-structured Interview Guide

Appendix 1

**Pre-Op Patients**

Tell us about your decision to use surgery as a means of treating LLS

What information and resources did you draw on when making this decision?

What resources did you find particularly useful?

We are interested in your thoughts on pre-operative consultation in helping you to prepare.

Help us to understand any concerns you might have going into the surgery.

Describe as best you can what an acceptable or satisfactory outcome for you (e.g. physical function, social, pain, work, quality of life).

Is there a difference between how you define being ‘recovered’ versus being ‘satisfied with your outcome’? What is the best-case scenario as an outcome of the surgery?

**Post-Op Patients**

Looking back, tell us about your decision to use surgery as a means of treating LLS

Can you tell me about your recovery process since the surgery.

Was the information and resources you received help this process? Was there additional information or that you would have found helpful? What resources did you find particularly useful?

Are there resources and information you are using now to assist with recovery?

We are interested in your thoughts on the operative consultations in helping you to prepare and recover.

Were there concerns you had going into the surgery? Tell us your thoughts on those concerns now. Are there concerns now that you did not anticipate?

Describe as best you can what an acceptable or satisfactory outcome is you (e.g. physical function, social, pain, work, quality of life).

Is there a difference between how you define being ‘recovered’ versus being ‘satisfied with your outcome’? What is the best-case scenario as an outcome of the surgery?
